# Compatibilization of Poly(Lactic Acid) (PLA)/Plasticized Cellulose Acetate Extruded Blends through the Addition of Reactively Extruded Comb Copolymers

**DOI:** 10.3390/molecules26072006

**Published:** 2021-04-01

**Authors:** Maria-Beatrice Coltelli, Norma Mallegni, Sara Rizzo, Stefano Fiori, Francesca Signori, Andrea Lazzeri

**Affiliations:** 1 Dipartimento di Ingegneria Civile ed Industriale, Università di Pisa, via Diotisalvi 2, 56126 Pisa, Italy; norma.mallegni@gmail.com (N.M.); sararizzo@outlook.com (S.R.); francesca.signori@unipi.it (F.S.); andrea.lazzeri@unipi.it (A.L.); 2 National Interuniversity Consortium of Materials Science and Technology (INSTM), c/o via Diotisalvi 2, 56126 Pisa, Italy; 3 Condensia Quimica, C/Junqueras 11-A, 08003 Barcelona, Spain; s.fiori@condensia.com; 4 Istituto per i Processi Chimico Fisici, IPCF-CNR, Area della Ricerca di Pisa, Via Moruzzi 1, 56100 Pisa, Italy

**Keywords:** rigid packaging, PLA blends, reactive extrusion, compounding, compatibilization effect

## Abstract

In the perspective of producing a rigid renewable and environmentally friendly rigid packaging material, two comb-like copolymers of cellulose acetate (AC) and oligo(lactic acid) OLA, feeding different percentages of oligo(lactic acid) segments, were prepared by chemical synthesis in solvent or reactive extrusion in the melt, using a diepoxide as the coupling agent and were used as compatibilizers for poly(lactic acid)/plasticized cellulose acetate PLA/pAC blends. The blends were extruded at 230 °C or 197 °C and a similar compatibilizing behavior was observed for the different compatibilizers. The compatibilizer C1 containing 80 wt% of AC and 14 wt% of OLA resulted effective in compatibilization and it was easily obtained by reactive extrusion. Considering these results, different PLAX/pAC(100-X) compounds containing C1 as the compatibilizer were prepared by extrusion at 197 °C and tested in terms of their tensile and impact properties. Reference materials were the uncompatibilized corresponding blend (PLAX/pAC(100-X)) and the blend of PLA, at the same wt%, with C1. Significant increase in Young’s modulus and tensile strength were observed in the compatibilized blends, in dependence of their morphologic features, suggesting the achievement of an improved interfacial adhesion thanks to the occurred compatibilization.

## 1. Introduction

Poly(lactic acid) (PLA), a recyclable and compostable thermoplastic polyester, produced from renewable resources, is currently proposed for many applications, also in the perspective of circular and green economy principles [1,2,3,4]. However, improvements in PLA performances, especially for specific applications, are still needed. Indeed, enhancement of thermal, mechanical resistance, and toughness, without affecting stiffness, is pivotal for PLA-based materials [5], especially for those suitable for rigid packaging [6,7,8]. The blending with rigid and thermally resistant polymers like for instance polycarbonate (PC) [9,10,11,12], poly(ethylene terephthalate) (PET) [13], and polyamides [14] was explored, but the resulting materials were not fully biodegradable and partially from fossil sources. Hence, the blending with rigid polymers obtained from renewable sources could be considered a better option in the packaging sector, where the short life cycle of the products needs the most environmentally correct management. 

Cellulose acetate (AC), a biodegradable cellulose ester, is a rigid material, obtained by acetylation of renewable cellulose, and it is currently used in a wide range of applications [15,16,17,18]. Plasticized AC (pAC) is also a much versatile material [19,20], with an improved melt processability [19], used, among other applications, in active food packaging [21]. Different approaches have been investigated to make cellulose acetate a thermoplastic material [22], and among them the blending with other thermoplastic polymers, such as PLA, has been investigated. Unfortunately, PLA and pAC are immiscible materials [23], and need therefore proper compatibilization to improve compounds properties, especially in terms of toughness. Different approaches have been reported on the compatibilization of PLA/AC blends, such as the preparation, by ring opening polymerization, of AC-g-PLA copolymers to be used as compatibilizers [24,25,26]. 

Very recently, Xu et al. reported the synthesis by ring opening polymerization (ROP) of a grafted AC-g-PLA copolymer which can act as a plasticizer for cellulose acetate-based materials [26], while Choi et al. reported the use of different AC-g-PLA compatibilizers in cellulose acetate/PLA compounds [27]. However, these polymers were produced via traditional chemical synthesis. Generally, this can result time consuming and not easily up scalable. Reactive extrusion (REX) refers to the use of screw extruders as chemical reactors to perform chemical reactions of polymers or polymerizable monomers. A reactive extrusion approach, aimed at producing copolymers in a continuous equipment, would be certainly more convenient on an industrial viewpoint [28]. The most important advantage of reactive extrusion is the absence of solvents as a reaction medium. Moreover, the reactive extrusion offers many possibilities for flexible and economic production of materials with specific characteristics because it is a continuous process with a narrow residence time distribution and it has a high flexibility also enabling the economic production of small amounts of specialty polymers [29]. Interestingly, Quintana et al. [30] have synthesized Grafted D/L lactide to AC copolymers by reactive melt processing, performing the ROP via reactive extrusion methodology (thus performing the polymerization inside the extruder), using AC with different degree of acetylation and they have evaluated these copolymers as AC/PLLA blend compatibilizer. Grafted copolymers increased the adhesion between AC and PLA phases, leading to finer morphologies and materials with enhanced toughness. In any case, this approach requires a reactive extrusion step consisting of a polymerization, generally requiring a high residence time and thus not easy to be realized with a high productivity in a general-purpose extruder, but requiring specific dedicated instrumental configurations. Reactive extrusion processes considering polymers or oligomers as starting materials, hence considering the modification of polymeric structure more than the full polymeric synthesis, are certainly easier to perform and control.

Recently, we investigated the reactive extrusion of PLA/pAC blends in the presence of triacetin using tetrabutylammonium tetraphenylborate as catalysts [24]. The catalyst promotes, during the melt processing, the in situ random transesterification reaction between AC and PLA to give a AC-PLA copolymer, which acts as a compatibilizer of the two immiscible phases, thus determining the significant improvements in the mechanical and impact properties of the prepared PLA/pAC blends. However, the reaction occurring at the interface is not specific and it is not easy to be controlled, as AC cross-linking was also promoted in dependence of the adopted processing temperature [24]. Hence, the preparation of a tailored copolymer in previous step, before the blending with PLA, can be a promising alternative. Interestingly, it should be reminded that the extrusion at the optimized temperature of 197 °C up to 25% of pAC showed the tendency of the pAC phase to form fibrillated structures, thus leading to the preparation of composites with a PLA matrix and dispersed pAC fibers, and thus with the enhanced impact properties typical of fibers reinforced composites.

The preliminary production of a compatibilizer based on AC-g-PLA comb copolymers, obtained by combining cellulose acetate with poly(lactic acid) chains and by using a coupling agent, through a reactive extrusion approach, was never attempted, but it can result much advantageous because of the use of cheap additives largely available on a commercial scale and because of the simple production procedure. In particular, lactic acid oligomers (OLA) are widely available on a commercial point of view as they are used as plasticizer in PLA [31,32,33,34] or PVC [35,36]. Thus, in this study, the chemical synthesis of the compatibilizer consisting of cellulose acetate and OLA is described. In fact, cellulose acetate-based copolymers, bearing oligo(lactic acid) as pendant chains were synthetized by both synthesis in solvent and reactive extrusion, and used as compatibilizers in PLA-based compounds containing plasticized cellulose acetate as the secondary phase. The mechanical behavior, in terms of tensile and impact resistance, were tested and rationalized by means of the study of compound compositions and microstructures. A final evaluation and interpretation regarding the copolymer addition in PLA or PLA/pAC blends onto melt fluidity, tensile, and impact properties as a function of blend composition was then proposed.

## 2. Results

### 2.1. Preparation of Compatibilizers Based on Comb Copolymers

**Figure 1:**
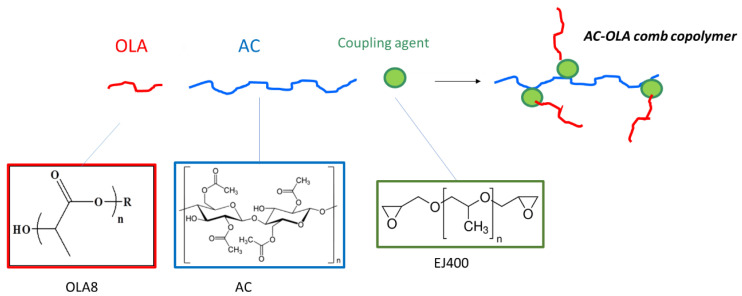
Strategy for the synthesis of AC-OLA comb copolymer by using a diepoxide (EJ 400) as coupling agent. In OLA8 formula *n* is in the range 3–15 and R- is derived from a high molecular weight linear alcohol.

The compatibilizers were designed as comb copolymers, having a cellulose acetate-based main chain and oligo(lactic acid) (OLA) pendant chains. A coupling agent consisting of a diepoxide (like EJ400) can react with hydroxyl groups of cellulose acetate and the terminal hydroxyl group of OLA (Figure 1). As OLA has only one reactive terminal, a brush structure is thus expected for the AC-OLA copolymer.

**Figure 2:**
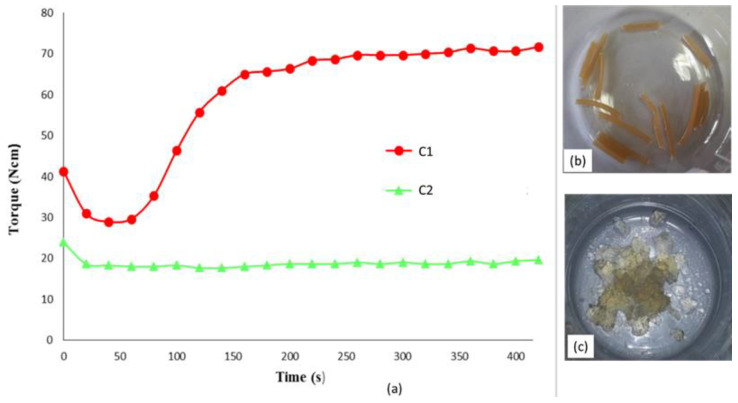
(**a**) Torque vs. time trend for the preparation of AC-OLA copolymer by mini-extrusion; picture of extruded strands (**b**) and the same after purification by double reprecipitation and drying (**c**).

The reaction was previously investigated in chemical laboratory performing it in solution. Two different compositions considering weight ratio AC/OLA/EJ400 80/14/6 and 57/30/13 (corresponding to Clab1 and Clab2 samples) were considered to control whether the final product structure could be dependent on the ratio between the AC polymer and the other low molecular weight reagents, whose ratio was maintained constant. Then the same reactions were carried out in mass in a mini-extruder, repeating the same compositions. The torque measurements, giving an indirect indication about melt viscosity, performed during the extrusion showed that C1 composition resulted in a final torque significantly higher than C2 (Figure 2a). This difference can be ascribed at the lower content of low molecular weight compounds in the C1 formulation. In the case of C2 composition, although the reaction of OLA8 and EJ400 with AC occurred, a significant amount of not reacted additives acted as plasticizer of the polymeric melt, decreasing its viscosity.

**Figure 3:**
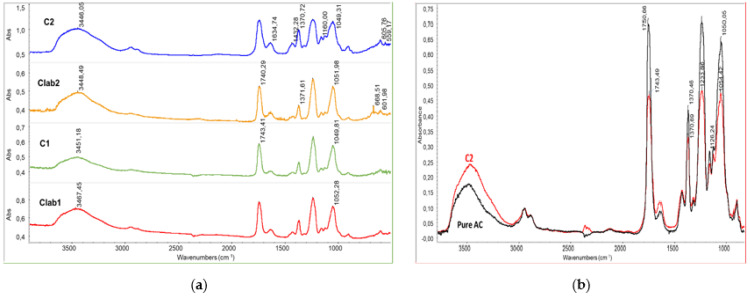
Infrared characterization of purified copolymers: (**a**) full infrared spectra of C1, C2, Clab1, and Clab2 samples; (**b**) comparison between the spectra of C2 and the pure cellulose acetate.

C1 and C2 differ in OLA content, having 14 and 30 wt % of OLA respectively. FT-IR spectra on purified final materials were performed to investigate whether the AC-OLA coupling reaction indeed occurred. The spectra of the purified AC-OLA copolymers produced in C1, C2, Clab1, and Clab2 trials (Figure 2a) show the peaks at 3450 cm^−1^ attributable to the stretching O-H of cellulose acetate structure, the peaks at 1740 cm^−1^, resulting from the stretching C=O of the ester bonds in both cellulose acetate and oligomeric lactic acid, and the peaks at 1370 cm^−1^ attributable to the bending CH. The peaks at 1233 cm^−1^ and at 1054 cm^−1^ can be attributed to the C-O stretching. The 1233 cm^−1^ peak is attributable to the C-O stretching of ester bonds. By comparing the infrared spectrum of pure AC and of one of the purified copolymers (Figure 3b), it can be evidenced, after normalizing on the peak at 2900 cm^−1^ related to the C-H stretching, the band of -OH at 3450 resulted increased, whereas the band at 1743 and 1233 cm^−1^ resulted decreased. These results can be attributed to the deacetylation occurring during the reaction [37]. Interestingly, this side reaction occurred in a similar extent during the synthesis in solvent or during the reactive blending procedure. In the case of the synthesis at 230 °C in miniextruder, the partial loss of acetic acid can be attributed to the typical degradation path of the plasticized AC, yet reported in literature [24]. In the case of chemical synthesis, the nucleophilic nature of the reagents and solvent can induce some breaking of the C-OCOCH_3_ linkages.

**Figure 4:**
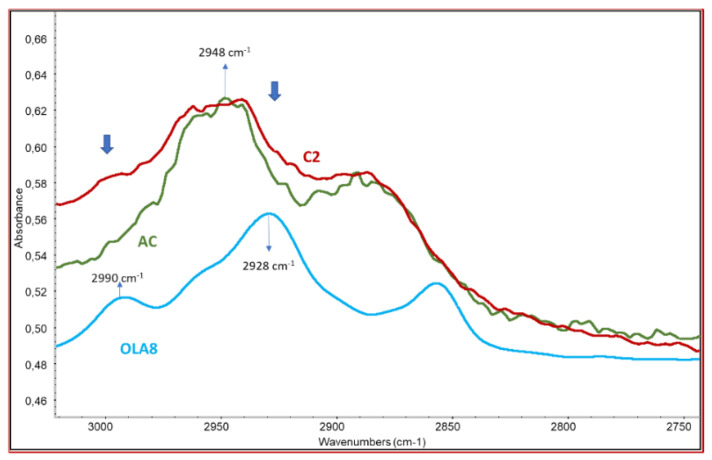
Infrared characterization of purified copolymer C2—comparison between the spectra of C2 and the pure cellulose acetate and oligo(lactic acid).

The peak of the carbonyl group in AC is at 1751 cm^−1^ as reported by Das et al. [38] and in good agreement with the spectrum of pure AC (Figure 3b). The spectra of all the samples show the band with a maximum at 1740–1743 cm^−1^, typical of aliphatic ester groups. It should be noticed that the pure OLA spectrum shows a C=O stretching band at 1750 cm^−1^, hence a decrease in the peak frequency can be observed in the C=O wavenumbers of OLA-AC samples that can be attributed to the presence of interactions between the ester groups in the modified AC samples or the occurrence of the modification of ester bonds because of the formation of new linkages [39], different from both those in pure OLA and in pure AC. As a significant fraction of samples resulted soluble in toluene (Table 1) we can deduct that there is only a slight modification due to the grafting of OLA on the AC chains. Another evidence of the occurrence of the grafting is related to the stretching of C-H range (Figure 4). In fact, the spectrum profile of the modified AC is different from the one of pure cellulose acetate because of the superposition of the grafted OLA signals, determining an increase of the absorbance at 2928 (asymmetric stretching of C-H in CH_2_ groups of OLA) and at about 2990 cm^−1^(asymmetric stretching of C-H in CH_3_ groups of OLA). 

Noteworthy, absorbance peak at 2948 cm^−1^, attributable to the CH stretching of AC, and the absorbance peak at 2928 cm^−1^, attributable to the CH stretching of OLA, resulted shifted in the prepared compatibilizers. In particular, a shift factor F was defined as reported in Equation (1).

(1) $$F=1-\frac{\left(R-R_{\mathrm{OLA}8}\right)}{\left(R_{\mathrm{AC}}-R_{\mathrm{OLA}8}\right)}$$

where, R is the height of the registered absorbance peak in the 2948–2928 cm^−1^ range for the two compatibilizers, R_OLA8_ is the height of the registered absorbance peak at 2928 cm^−1^ and R_AC_ is the height of the registered absorbance peak at 2948 cm^−1^. Since F = 0 for pure AC, and F = 1 for pure OLA8, expected F values are in the 0–1 range for compatibilizers when the coupling reaction has occurred. Results are reported in Table 1.

All the purified copolymers showed an F value between 0.47 and 0.6, indicating the occurrence of the grafting of OLA8 onto the AC chain. The values obtained by the reaction performed in the laboratory were similar to those obtained by reactive extrusion.

Interestingly, C1 and C2 returned similar F value, i.e., in the 0.45–0.60 range, suggesting that in both cases the reaction between AC and OLA8 indeed occurred even when the amount of low molecular weight additives introduced in the extruder was 20% by weight. This condition of processing can be particularly reliable, reasonably also in a scaling up industrial perspective.

### 2.2. Compatibilization Effectiveness of AC-OLA Comb Copolymer

Compatibilizers C1, Clab1, and C2, as obtained after preparation and thus without any purification, were compounded with PLA/pAC blends to test how the two different obtained compatibilizers influence the final properties of the blend.

A ratio 4/1 of pAC/compatibilizer blends was prepared preliminarily at 230 °C and then blended with PLA at 197 °C in a second step. This preliminary step was performed to disperse in a homogenous way the C1 copolymer in the pAC, in view of the successive blending step with PLA, considering that the copolymer should act as interfacial agent of all the pAC phase with the PLA matrix. The preliminary pre-blending was an important step as it could be observed by comparing the torque trend of plasticized AC with one of the plasticized AC containing 20% of C1 or Clab1. A consistent increase in torque with respect to the pAC can be observed and can be attributed to the capability of the partially unreacted EJ400 to further react with the pAC at 230 °C determining some occurrence chemical crosslinking. The extraction [40] of finely grinded pAC/C1 and pAC/Clab1 obtained at 230 °C with a mixture of acetone/water 90/10 (in which pAC resulted fully soluble), resulted in a residue of 8 and 11% by weight respectively, indicating the partial crosslinking of the pCA in the presence of the copolymer.

**Figure 5:**
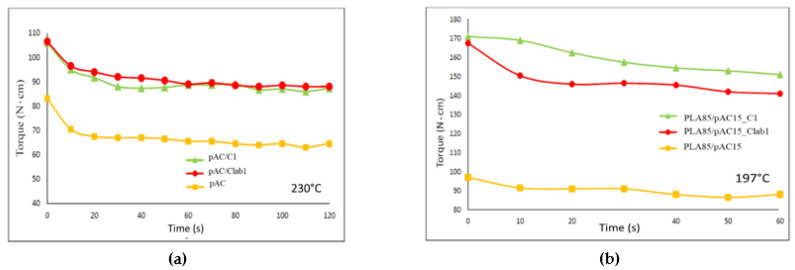
Torque vs. time trends: (**a**) extrusion of the plasticized AC with C1 or Clab1 (pre-blending) at 230 °C; (**b**) extrusion of blends containing 85% by weight of PLA at 197 °C [24].

Moreover, this pre-blended pAC-based blend was extruded with PLA to obtain PLA85/pAC blends containing 5% of C1 or Clab1. In good agreement with the preliminary observation (Figure 5a), the torque of the obtained blends containing C1 or Clab1 compatibilizer was higher than one of the reference, uncompatibilized blend (Figure 5b).

The obtained blends were then characterized in terms of their tensile and impact properties. The elastic modulus decreased by more than 25% by adding the compatibilizers, whereas the tensile strength and elongation at break did not change significantly. The Charpy Impact Strength slightly increased but only by adding the compatibilizer C1 (Table 2).

In general, C1 and Clab1 produced similar results, in agreement with a similar structure and reactivity.

**Figure 6:**
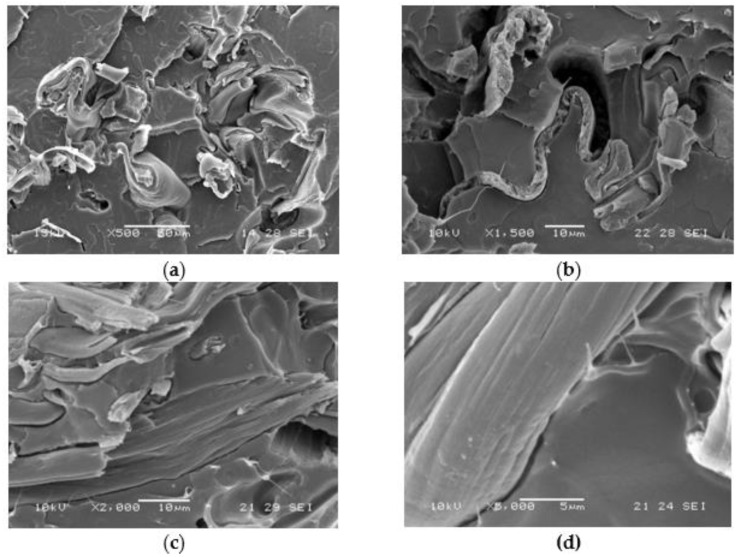
SEM micrographs obtained on cryo-fractured blends: (**a**) PLA85/pAC15 at 500× magnification; (**b**) PLA85/(pAC+Clab1)15 at 1500× magnification; (**c**) PLA85/(pAC+C1)15 at 2000× magnification; (**d**) PLA85/(pAC+C1)15 at 2000× magnification (detail of the pAC/PLA interface).

The samples in the form of extruded strands pieces were dissolved in a mixture of acetone/water 90/10 that can dissolve AC. A solid residue was obtained, indicating that PLA, insoluble in the solvent and main component of the blend, is the continuous phase. From the reference blend only 11% could be extracted. A dispersed morphology containing pAC in the form of fibers could be revealed in these blends [24]. The extractable material increased when the compatibilizers were used in agreement with a slight increase in the presence of AC grafted to OLA. However, the phase distribution was not significantly affected by the addition of the compatibilizers and remained characterized by dispersed fibrillated pAC in the PLA matrix. A clear improvement of adhesion between the two polymeric phases by using C1 could be noticed by SEM analysis (Figure 6) because the interface between the pCA fibrous minor phase and the PLA matrix could be revealed with more difficulty (Figure 6c,d) than in the reference PLA85/pAC15 blend (Figure 6a). As the surface was cryofractured in liquid nitrogen before the analysis, in the not compatibilized sample the interface resulted brittle and the PLA matrix was often found as detached from the fibrous phase during the breaking (Figure 6a,b). On the contrary, the PLA matrix was found not broken and detached in the compatibilized blend (Figure 6c,d).

The decreased thickness of the fibrils dispersed phase in the blend compatibilized by C1 determined an improved capacity of the matrix to yield, thus better dissipating the energy during the impact test. This can be considered a good effect of compatibility.

**Figure 7:**
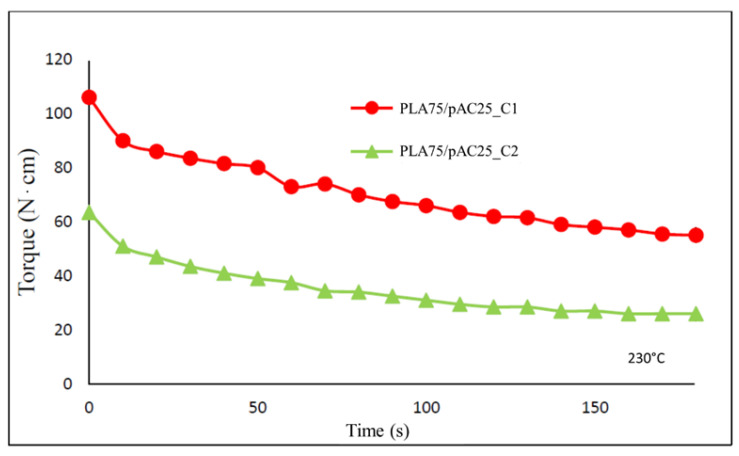
Torque vs time trends for the extrusion of PLA75/pAC25 blends compatibilized with C1 or C2.

PLA75/pAC25 blend, presenting the pAC phase partially continuous [24], was selected as reference to compare C1 and C2 that were added at 5 wt%. The extrusion was performed at 230 °C. Noteworthy, the final torque values resulted quite different in the two cases. Indeed, final torque values resulted almost double (55 ± 1 N*cm) for sample PLA75/pAC25_C1 with respect to sample PLA75/pAC25_C2 (26 ± 4 N*cm). This behavior can be rationalized considering the different viscosity due to the different pAC/OLA8 molar ratio in the two compatibilizers (Figure 7). However, similar slope was found in the two cases, suggesting that comparable processing features occurred during the blending.

The mechanical properties of the blends were then measured (Table 3).

**Figure 8:**
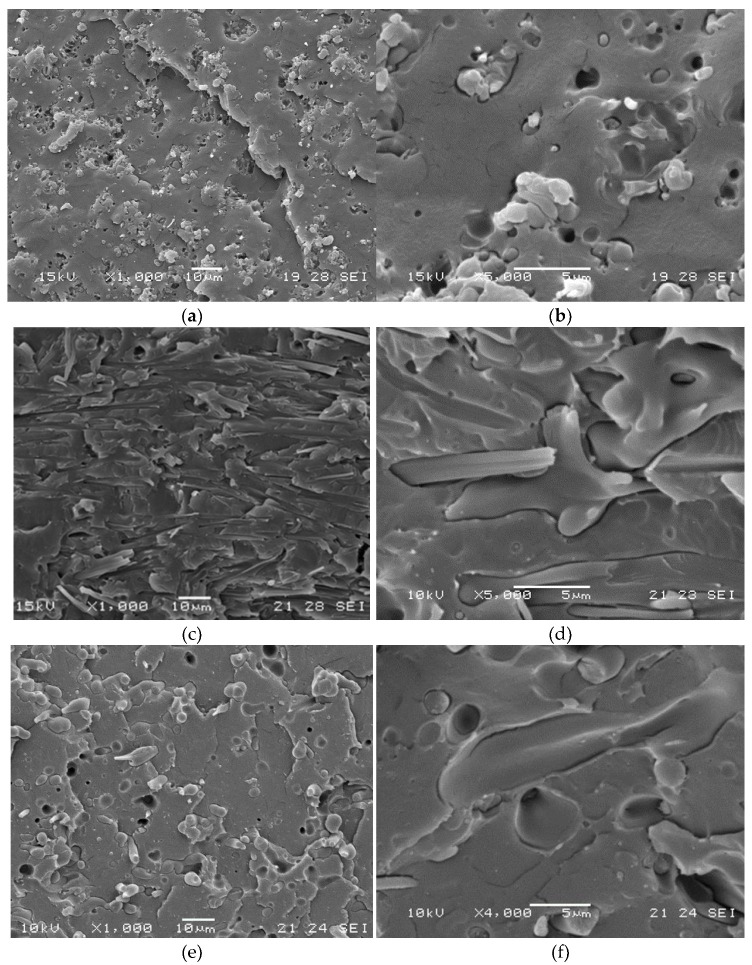
SEM micrographs obtained on cryo-fractured blends of samples PLA75/pAC25_C1 and PLA75/pAC25_C2 compounded at 230 °C: (**a**) PLA75/pAC25 at 1000× magnification; (**b**) PLA75/pAC25 at 5000× magnification; (**c**) PLA75/pAC25_C1 at 1000× magnification; (**d**) PLA75/pAC25_C1 at 5000× magnification; (**e**) PLA75/pAC25_C2 at 1000× magnification; (**f**) PLA75/pAC25_C2 at 4000× magnification.

Interestingly, the addition of C1 and C2 resulted in significantly increased E-Modulus and stress at break of the prepared compounds, suggesting that the compatibility between pAC and PLA indeed improved. Noteworthy, the two compatibilizers returned comparable Young’s Modulus values, but C1 resulted significantly more efficient in the improvement of the stress at break of the prepared compounds. The low amount of OLA8 in C1 is sufficient to guarantee compatibility between cellulose acetate and PLA, without affecting the mechanical properties of the cellulose acetate main chain, mainly in terms of strength. Accordingly, SEM analysis returned a more homogenous, fiber like morphology for sample PLA75/pAC25_C1 with respect to that observed for sample PLA75/pAC25_C2, where more spherical dispersed particles can be observed (Figure 8).

However, SEM analysis seems to indicate the presence of a co-continuous morphology in both PLA75/pAC25_C1 and PLA75/pAC25_C2 cases, thus explaining the loss in impact resistance of the analyzed blends.

These results suggest that the prepared C1 and C2 compatibilizers are effective in PLA/AC blends and they behave in a similar way. As the production of C1 is simple, this compatibilizer was kept into account for further investigating blends produced at 197 °C.

### 2.3. Properties of PLA Blends Containing C1 and pAC

**Figure 9:**
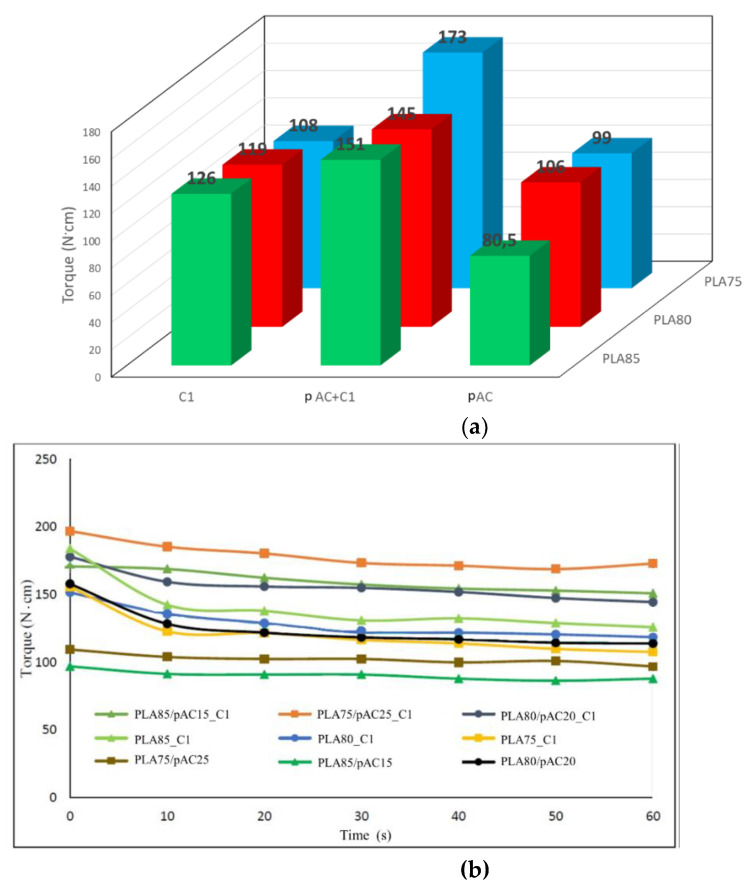
Torque behavior of blends with 75, 80 and 85% of PLA and pure pAC, pAC/C1 in 4/1 ratio or pure C1: (**a**) final torque data plotted as a function of PLA content and C1 content; (**b**) torque vs. time trends.

Therefore, further PLA/pAC compositions have been investigated, all having PLA as the matrix component and 15, 20, and 25% of pAC. This composition range was selected as it was found that significant morphology variations, allowing a wide properties modulation could be obtained in this narrow composition range [24]. Moreover, for each composition, C1 was added to pure PLA sample, to investigate whether peculiar effects on the matrix component occurred. Note that all the blends were compounded at 197 °C instead of 230 °C as previously performed, thus limiting polymer degradation upon processing and promoting the pAC fibrillation in the PLA matrix [24]. Detailed compositions of the investigated blends are reported below. As yet noticed in Figure 4, the blend of pAC and C1 has a higher torque than pAC, hence a significant increase of torque was observed in all the PLAX/pAC(100-X) C1 blends with respect to corresponding PLAX/pAC(100-X) blends. Note that increasing the amount of pAC up to 25 wt% determines an increase in the torque values (Figure 4a and Supplementary Table S1). On the other side, the substitution of the pAC with C1 in PLA blends having the same composition determines a drop in the torques values, suggesting a decreased viscosity of C1 with respect to pAC mixed with C1, in agreement with the trend observed in Figure 5a. The torque vs. time trends showed constant value, indicating that during the extrusion no significant degradation or cross linking occurred in the melt blends (Figure 9b).

**Figure 10:**
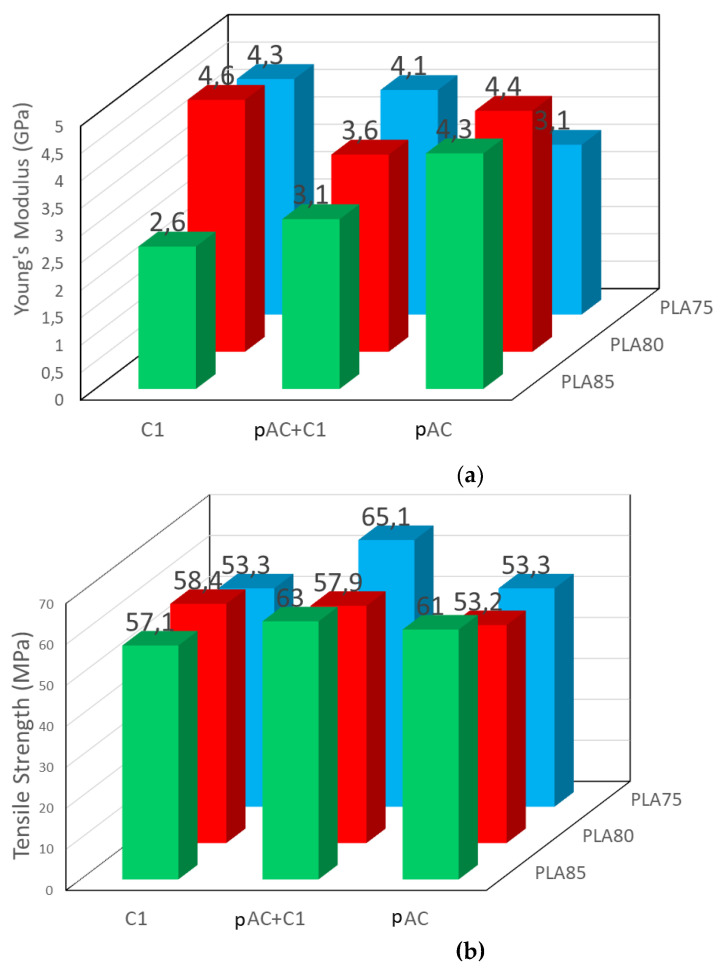
Tensile properties of blends with 75, 80 and 85% of PLA and pure pAC, pAC/C1 in 4/1 ratio or pure C1: (**a**) Young’s modulus; (**b**) tensile strength.

Mechanical properties of the prepared compounds measured by tensile tests are reported in Figure 10 and Supplementary Tables S2 and S3. For each composition, three samples were tested and compared: the uncompatibilized blend (PLAx/pAC(100-X)), the compatibilized one (PLAx/pAC(100-X)_C1), and, for further comparison, the one having PLA at the same composition and C1 as the second phase (PLAx_C1). These latter blends were prepared to maximize the presence of the copolymer, and thus interfacial bonding, in the PLA-based blends.

Regarding the Young’s Modulus, the blends containing 85% of PLA showed a decreasing trend by adding increasing amount of C1 in the blend. This composition is characterized by a dispersed morphology [24], hence the compatibilizer induces a decrease of elongated dispersed phase dimension, thus favoring matrix deformation. Correspondingly, the tensile strength is maintained constant, as the higher deformability can allow a better energy dissipation during tensile tests.

The blend containing 75% of PLA had an opposite trend. In fact, this composition is characterized by a co-continuous morphology [24]. It means that the pAC phase consists of a continuous, interconnected phase. Hence the improved compatibilization cannot significantly decrease the phase dimensions, that remains continuous. Then, the most evident effect of compatibility is the presence of bonds at the interface, resulting in an increased stiffness and a decreased deformability of the matrix. This effect was observed in several cases, as an example in polyamide blends [41,42], polyester [43], or polycarbonate [9,44] based blends. Interestingly, the addition of C1 to pAC gave the best value of tensile strength, probably because of the achievement of a good balance between increased stiffness and deformability of the matrix. The blend containing 80% of PLA showed an intermediate behavior, much dependent on the content of C1 in the blend. 

Interestingly, the stress at break increased as a consequence of the addition of the compatibilizer, which therefore resulted effective in the improvement of the PLA/AC interface. In particular, the addition of C1 at 5% seemed a very good strategy to have a significant increase of tensile strength, especially in the blend containing PLA at 75% by weight. In general, the blend containing only C1 had a higher Young’s Modulus and lower tensile strength, in agreement with a more extended presence of interfacial bonds due to compatibilization. This result suggests that indeed C1 acted as a compatibilizer. The compatibilizer, having an AC backbone and oligomeric lactic acid lateral chain (Figure 1) would preferentially stay at the interface (between the PLA and AC phase). Hence in this small region linkages between the cellulose acetate phase and the PLA phase will be present, determining an increased resistance when the material is stressed with respect to uncompatibilized blends. These linkages correspond to the EJ400 “bridges” (Figure 1 green circles). These bridges will be obviously more concentrated in the blends containing only C1, where the copolymer is more concentrated.

**Figure 11:**
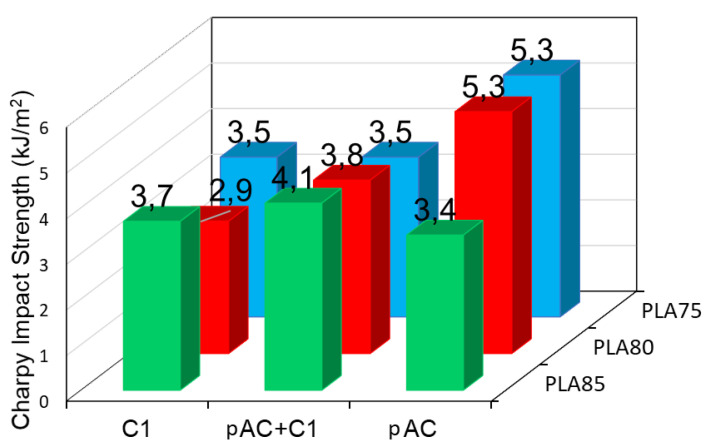
Charpy impact strength of the blends with 75, 80 and 85% of PLA and pure pAC, pAC/C1 in 4/1 ratio or pure C1.

Moreover, the addition of the compatibilizer decreased the impact resistance of the compatibilized blends (Figure 11), with the exception of blend containing 85% of PLA, where a slight increase in impact properties was observed with respect to the reference, uncompatibilized blend. Again, the results are the consequence of the different phase morphology of the blends at different composition. For the 85% blend, where the pAC is dispersed in the PLA matrix, the compatibilizer allowed the formation of elongated pAC fibers of lower dimension, thus allowing a better deformation (possible voids formation) of the matrix and energy dissipation. On the contrary, when the starting matrix was partially co-continuous (as in the case of blends having at least 20% of pAC), the increased stiffness and thus the limited possibility of voids generation decreased the impact strength. 

Dissolution tests were performed on the prepared PLAX/pAC(100-X)C1 blends extruded strand, using a 90/10 acetone/water solution in which AC was soluble while PLA resulted in a solid residue of the same shape of the extruded strand. These results (Table 4) indicated that the PLA was a continuous phase in all the blends. 

In blends up to 20% of pAC content, a higher amount was extracted than in the reference uncompatibilized blend, indicating that when the content of AC is higher than 20%, a partial continuity of the AC phase is obtained. As this phase incorporate some dispersed PLA, the extraction is much more effective. However, comparing the data with the corresponding reference blends it can be noticed that the extracted mass was higher than in the reference, for uncompatibilized blends up to 20% of pAC content, but above the opposite can be observed. These results suggest that for up to 20% of pAC a dispersed morphology was obtained, whereas above this pAC content a partial continuous pAC phase is present. In the blends up to 20% of pAC the effective presence in compatibilized blends of polyester chains, highly interacting with AC and thus soluble in the extraction solvent, can also explain the obtained results. The PLA80/pAC20_C1 is reasonably co-continuous, because of the high amount of extracted mass, higher than the pAC content in the blend. 

If we consider the PLA75/pAC25, having a partial continuous pAC phase, the addition of the compatibilizer determined a decrease in extractable pAC, probably because of the lower dimension of the interconnected filaments in the co-continuous structure, making the pAC less extractable. This agrees with the compatibility action exerted on co-continuous morphologies [45]. A number of studies [46,47,48] have also shown that premade di- or tri-block copolymers tend to decrease the characteristic phase size and in narrowing the co-continuity range of immiscible polymer blends, which is attributed to its ability of suppressing droplet coalescence.

## 3. Discussion

The structure and effectiveness of the compatibilizers obtained by chemical synthesis or by extrusion, starting from cellulose acetate (AC), EJ40 and oligomeric lactic acid (OLA), resulted similar and well defined. Copolymers having a cellulose acetate partially deacetylated structure and low amount of OLA side chains were obtained. Moreover, the change in composition of C1 and C2 (where the ratio between AC and liquid additive was 80/20 and 60/40 respectively) did not determine any significant change in properties, thus indicating that C1 sample, easier to be obtained, is the best option.

**Figure 12:**
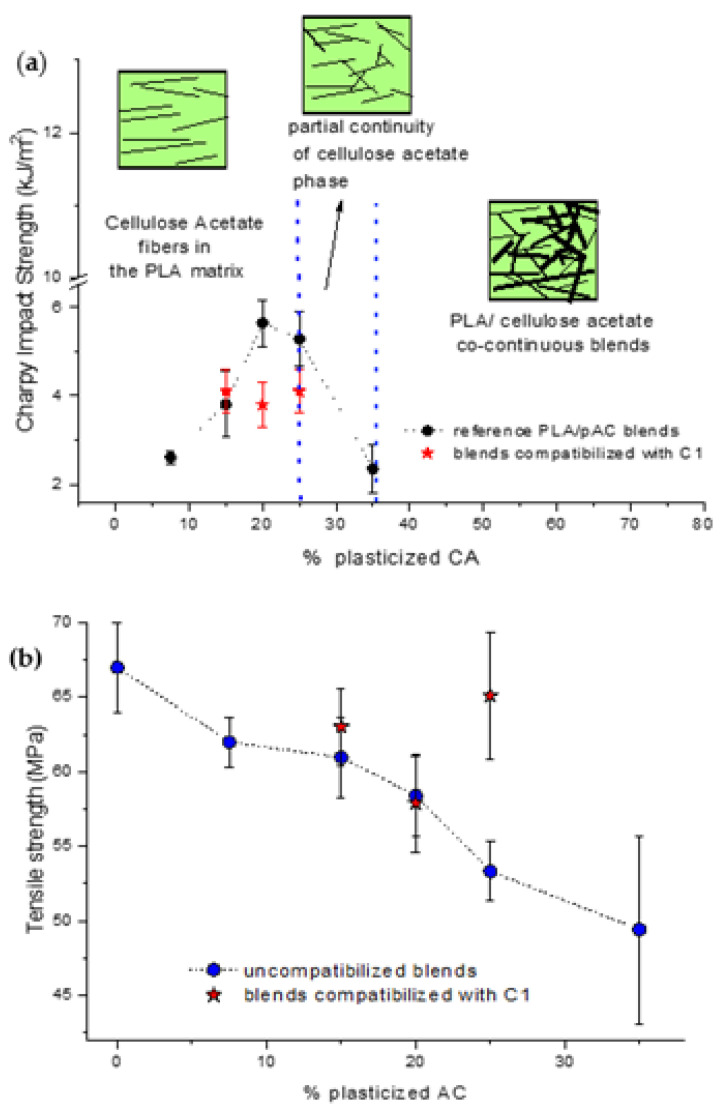
Comparison with the properties of reference blends PLA as a function of pAC content (% by weight). (**a**) Tensile strength; (**b**) Charpy impact strength.

The present work keeps into account the results reported in a previous work [24], where the properties of the PLA/pAC blends were studied in a wide composition range. These investigations, based on the study of phase morphology and properties of the blends, showed that up to 20% by weight the composites produced at 197 °C resulted PLA matrix composites reinforced with pAC fibers as a dispersed phase. Between 20 and 25% the presence of partial continuity of the pAC phase was evidenced and above 25% the blends were fully co-continuous. This change in the phase morphology as a function of composition resulted in a peculiar shape of the Charpy impact strength curve (Figure 12a) as a function of pAC content, showing a local maximum for the blends with 20% and 25% of pAC. The formation of fibrillated structure for pAC in the PLA matrix was thus evidenced, leading to a morphological structure typical of fiber-reinforced composites. This peculiar, dispersed morphology was justified by considering the high viscosity ratio of the dispersed phase vs. the matrix [24,48]. By increasing this ratio, the tendency to obtain a fibrillated morphology and a co-continuous morphology, originating from the interconnection of phase fibrils [49,50,51,52], is favored.

In a fiber-reinforced composite, the compatibilization can result in an improvement of stiffness but in a decrease in ductility. In this kind of composite, it is frequently reported that the addition of the compatibilizer determined an increase in the interfacial adhesion, that negatively affects the capability of the material to absorb energy during breakage. In fact, if from one side, a good adhesion is responsible for the strength at break increment, on the other hand, a toughness improvement is achieved where low adhesion is obtained [53]. The capability of a material to absorb energy by void formation is in fact discouraged by a strong interfacial adhesion [54,55].

In good agreement, the good compatibility effect showed by this comb like compatibilizer was effective in determining the possibility to increase the elastic modulus and the tensile strength because of the increased rigidity of the macromolecular structure ascribable at the linkages present at the PLA/pAC interface. Especially for the PLA blends containing 25% of pAC, a significant increase in the elastic modulus and in the stress at break were observed. The fibrillated morphological structure was not significantly affected by the addition of the compatibilizer, as evident from the morphological analysis (Figure 6). 

The results are also in agreement with the tendency of the compatibilized blends to have a co-continuous morphology because of the increased viscosity of the pAC phase, favoring fibrillation and also co-continuity. This can explain the differences in properties observed between the PLAx_C1 and the PLAx/pAC(100-x)_C1 blends. In fact, these latter have a pAC phase more viscous and this favors the development of a fibrillated and co-continuous morphology with respect to the PLAx_C1 blends.

## 4. Materials and Methods

### 4.1. Materials

Poly(lactic acid) (PLA), purchased from NatureWorks LLC (Minnesota, MN, USA), was Ingeo 2003D Extrusion Grade, having a nominal average molecular weight (M_w_) of 199590 and a density of 1.24 g/cm^3^.

Pure cellulose acetate (AC) and triacetine plasticized cellulose acetate (pCA) with a tracetine content of 20 wt% were purchased by GIBAPLAST (Varese, Italy). 

Oligo(lactic acid) (GLYPLAST OLA8) was purchased by Condensia Quìmica SA (Barcelona, Spain). It is a yellowish viscous liquid having a density of 1.11 g/cm^3^, a glass transition of −52.9 °C, and a viscosity of 22.5 mm^2^/s. This oligopolymer has a Mw of 1400 g/mol and it is thermally stable up to 188 °C in air.

Poly(propylenglycol) diglycidyl ether, Glyether^®^ Resin, (EJ-400) from Jsi Co., Ltd., Pyeongtaek, Gyeonggi, Korea, was used as received [density 1.21 g/cm^3^, molecular weight: 305 g/eq].

### 4.2. Preparation of the Compatibilizers by Chemical Synthesis

The compatibilizer produced in the laboratory was obtained by means of a solvent reflux reaction. The powdered acetate was solubilized in dioxane at a temperature of about 50 °C to facilitate solubilization and decrease its time, subsequently OLA8 was added and then, drop by drop, EJ400. After adding all the reagents, the solution was heated up to the boiling temperature of the dioxane (about 105 °C, at atmospheric pressure) and kept under stirring under the reflux of the solvent for two hours. Subsequently, the solvent was eliminated by evaporation in Rotavapor thus obtaining a residue that was left for about 24 h in a ventilated oven at 60 °C to complete drying. For the formulation of compatibilizer 1 (Clab1) a total of 5 g of reagents were used. C1 lab composition was AC/OLA8/EJ400 80/14/6 by weight. C2Lab composition was AC/OLA8/EJ400 57/30/13 by weight. Crude Clab1 and Clab2 materials were purified by double precipitation in toluene from a dioxane solution; the recovered materials were further washed with toluene and then dried at 60 °C.

### 4.3. Preparation of the Compatibilizers by Reactive Extrusion

The compatibilizers, C1 and C2, were prepared by reactive extrusion by means of a Minilab II HaakeTM Rheomex CTW5 conical twin screws extruder at 230 °C. The screw rate was 100 rpm, and the extrusion was 7 min long. Table 5 reported the feed compositions of the two compatibilizers, that was identical to the one of C1lab and C2lab respectively.

Crude C1 and C2 materials were purified by precipitation in toluene from a dioxane solution, the recovered materials were further washed with toluene and then dried at 60 °C.

The C1 and C2 materials, as recovered from miniextruder, were blended with pAC in ratio 1:4 by weight at 230 °C for preparing PLAX/pAC(100-X)_C1 or PLAX/pAC(100-X)_C2 blends. This procedure was followed because of its easy potential upscale.

### 4.4. Compatibilizers Characterization

FT-IR spectra were recorded using a Thermo Nicolet 380 FT-IR spectrophotometer, in the 4000–400 cm^−1^ range. The copolymer was dissolved on chloroform and deposited on KBr disks. The transmission spectra were recorded after solvent evaporation. OMNIC FT-IR software was used for spectra elaboration.

The pAC, the pAC containing C1 and C2 in ratio 4:1 (see Section 4.3) were finely ground and extracted with acetone/water at 50 °C for 6 h to determine the residual fraction. Each extraction was replicated.

### 4.5. Compounds Preparation

Poly(lactic acid) PLA, plasticized cellulose acetate pAC, oligo(lactic acid) OLA8, C1, C1lab and C2 (coming from the preliminary synthesis described in Section 4.2 and Section 4.3) were dried for 24 h at 60 °C in a ventilated oven before extrusion.

PLA-based compounds (Table 6) were extruded in a MiniLab II HaakeTM Rheomex CTW5 conical twin screws extruder (Thermo Fisher Scientific, Waltham, MA, USA) at 197 or 230 °C. The screw rate was 100 rpm, and the extrusion was 1 min long. Directly after the extrusion, the molten materials were transferred through a preheated cylinder to the HaakeTM MiniJet mini-injection moulder (Thermo Fisher Scientific, Waltham, MA, USA) to obtain Haake type III specimens (total length 87 mm, total width 10 mm, thickness 1.5 mm) that were used for tensile measurements. Torque measurements were performed by using the MiniLab II HaakeTM Rheomex CTW5 conical twin screws extruder at 197 or at 230 °C. The extrusion was monitored for up to 60 s to obtain the trend as a function of the extrusion time. Compound compositions are reported in Table 6.

### 4.6. Compounds Characterization

Tensile tests were performed at room temperature at a crosshead speed of 10 mm/min by means of an Instron 4302 universal testing machine (Canton, MA, USA), equipped with a 10 kN load cell and interfaced with a computer running the TestWorks 4.0 software (MTS Systems Corporation, Eden Prairie, MN, USA).

Impact tests were performed on V-notched specimens (width:10 mm, length: 80 mm, thickness: 4 mm, V-notch 2 mm at 45°) using a 15 J Charpy pendulum on an Instron CEAST 9050 (CEAST, Torino, Italy), equipped with DAS 8000 junior for data recording at a frequency of 1000 kHz. The standard method ISO179:2000 was followed. For each blend, at least ten specimens were tested at room temperature.

The morphology of the blends was studied by scanning electron microscopy (SEM) using a JEOL JSM-5600LV (Tokyo, Japan), by analysis of the cryo-fractured surfaces that were previously sputtered with gold.

Solubility tests were performed by stirring compound samples at room temperature for 24 h in a 90/10 acetone/water solution. Residual samples were washed twice with fresh solvent and then dried at 60 °C for 24 h. Amounts of lost matter were determined gravimetrically.

## 5. Conclusions

Comb-like copolymers of cellulose acetate (AC) and oligo(lactic acid) OLA, were prepared both by synthesis in solvent and by reactive extrusion, using a diepoxide as the coupling agent. The copolymers were characterized by infrared spectroscopy after purification and some deacetylation as well as slight grafting of OLA on the AC was observed in a similar extent in all the produced samples, despite of they were produced by synthesis in solvent or reactive extrusion and despite of they were obtained by feeding different OLA and diepoxide contents in the reaction. The prepared materials were used, after a preliminary pre-blending with plasticized cellulose acetate in a 1:4 weight ratio, as compatibilizers for poly(lactic acid)/plasticized cellulose acetate (PLA/pAC) blends, prepared at different temperatures (197 and 230 °C) showing a similar mechanical and morphologic behavior, indicating the occurrence of compatibilization because of increased adhesion between the PLA and pAC phase and because of the significant modification of properties due to the presence AC-OLA linkages in the interfacial region of the blend.

Considering these results, different PLA compounds with variable PLA content and containing C1 were prepared at 197 °C. Two series of blends were extruded: those containing a mixture 1:4 by weight of C1 and pAC and those containing only C1. The latter blends were prepared to maximize the concentration of AC-OLA copolymer in the binary system. Significant increase in Young’s modulus and tensile strength were observed in many compatibilized blends, in dependence of their morphologic features, suggesting the achievement of an improved interfacial adhesion owing to the occurred compatibilization, especially in the blends containing only C1, where the AC-OLA copolymer was more concentrated and thus available in the interfacial region. The data were also discussed, considering the morphology evolution of these blends as a function of composition, thus extracting interesting suggestions for further modulation of PLA/pAC blends properties.

As these blends can represent a good alternative to rigid and stiff PLA-based blends, the application of reactive extrusion methodologies for their improvement can result in a promising strategy for accelerating the replacement of fossil-based not biodegradable materials with renewable and biodegradable ones in several applications, including rigid packaging.

## Figures and Tables

**Table 1:** FT-IR analysis of C1 and C2, the AC/OLA based prepared compatibilizers. Compositions of C1 and C2 are reported in the experimental part.

Sample	Residue (wt.%)	λ (cm^−1^)	R (H_2948_/H_2928_)	F (Equation (1))
AC	-	2948.25	1.17	0
OLA8	-	2928.04	0.89	1
Clab1	64	2945.20	1.01	0.57
Clab2	51	2945.42	1.01	0.6
C1	80	2946.62	1.02	0.55
C2	56	2942.81	1.04	0.47

**Table 2:** Mechanical and impact properties of the PLA85/pAC15 blends compounded with Clab1 and C1.

Entry	E (GPa)	Tensile Strength (MPa)	Elongation at Break (%)	Charpy Impact Strength (KJ/m^2^)	Wt% Residue to Acetone/Water
PLA85/pAC15	4.3 ± 1.3	61 ± 3	2.1 ± 0.6	3.4 ± 0.4	89
PLA85/pAC15_Clab1	3.2 ± 0.5	56 ± 6	2.2 ± 0.6	3.4 ± 0.3	87
PLA85/pAC15_C1	3.1 ± 0.3	63 ± 3	2.6 ± 0.5	4.1 ± 0.5	85

**Table 3:** Mechanical properties of the PLA75/pAC25 blends compounded with C1 and C2.

Entry	E (GPa)	Tensile Strength (MPa)	Elongation at Break (%)	Charpy Impact Strength (KJ/m^2^)
PLA75/pAC25	3.4 ± 0.9	48.2 ± 4.5	1.5 ± 0.3	4.1 ± 0.7
PLA75/pAC25_C1	4.2 ± 0.5	64.9 ± 4.7	1.9 ± 0.1	2.2 ± 0.5
PLA75/pAC25_C2	4.5 ± 1.3	55.7 ± 4.0	1.6 ± 0.3	1.9 ± 0.3

**Table 4:** Results related to dissolution tests performed onto extruded strands of the blends with 75, 80, and 85% of PLA and pure pAC, pAC/C1 in 4/1 ratio.

Entry	Residual Mass (wt.%)	Extracted Mass (wt.%)
PLA85/pAC15	88.6 ± 0.6	11.4 ± 0.6
PLA85/pAC15_C1	85.1 ± 0.5	14.9 ± 0.5
PLA80/pAC20	96.0 ± 0.7	4 ± 0.7
PLA80/pAC20_C1	75.4 ± 0.5	24.6 ± 0.5
PLA75/pAC25	80.3 ± 0.4	19.7 ± 0.4
PLA75/pAC25_C1	84.3 ± 0.5	15.7± 0.5

**Table 5:** Composition of C1 and C2 compatibilizers.

Entry	C1 (wt%)	C2 (wt%)
AC	80	57
OLA8	14	30
EJ-400	6	13

**Table 6:** Composition wt% of the prepared compounds.

Entry	AC wt%	PLA wt%	C1 wt%	C2 wt%	Clab1	Extrusion Temperature (°C)
PLA75/pAC25_C1	20	75	5			230
PLA75/pAC25_C2	20	75		5		230
PLA75/pAC25	25	75				230
PLA75/pAC25_C1	20	75	5			197
PLA75/pAC25	25	75				197
PLA75_C1		75	25			197
PLA85/pAC15_C1	10	85	5			197
PLA85/pAC15_Clab1	10	85			5	197
PLA85/pAC15	15	85				197
PLA85_C1		85	15			197
PLA80/pAC20_C1	15	80	5			197
PLA80/pAC20	20	80				197
PLA80_C1		80	20			197

## Data Availability

Data is contained within the article or Supplementary Materials.

## Supplementary Materials

Table S1: Detailed composition of the investigated PLA/pAC blends and Torque values with standard deviation; Table S2: Mechanical properties by tensile tests and impact resistance of PLA/Pac.; Table S3: Tensile data of each tested specimen and average values of tensile properties with standard deviation; Figures S1 and S2: Overlay of 5 Stress Strain curves of PLAX_C1 andPLAX_pAC(100-X)_C1 blends.

## Sample Availability

Samples of the films are available from the authors.
