# Supplementary material for: Compatibilization of Poly(Lactic Acid) (PLA)/Plasticized Cellulose Acetate Extruded Blends through the Addition of Reactively Extruded Comb Copolymers
Source: Molecules. 2021 Apr 1;26(7):2006. doi: 10.3390/molecules26072006 (PMC8037935; doi:10.3390/molecules26072006)
Supplement: Supplementary file 1 [file molecules-26-02006-s001.pdf]

Compatibilization of poly(lactic acid) (PLA)/ plasticized cellulose acetate extruded blends through the addition of reactively extruded comb copolymers

Maria-Beatrice Coltelli, Norma Mallegni, Sara Rizzo, Stefano Fiori, Francesca Signori and Andrea Lazzeri

Supplementary data

Table 1: detailed composition of the investigated PLA/pAC blends and Torque values with standard deviation

Entry	pAC wt%	PLA wt%	C1 wt%	Torque (N*cm)
**PLA85/pAC15**	**15**	**85**	**-**	**80 ±15**
**PLA85/pAC15_C1**	12	85	3	151 ± 3
**PLA85_C1**	-	85	15	126 ± 1
**PLA80/pAC20**	20	80	-	106 ± 6
**PLA80/pAC20_C1**	16	80	4	145 ± 5
**PLA80_C1**	-	80	20	119 ± 7
**PLA75/pAC25**	25	75	-	99 ± 15
**PLA75/pAC25_C1**	20	75	5	173 ± 13
**PLA75_C1**	-	75	25	108 ± 20

Table 2: Mechanical properties by tensile tests and impact resistance of PLA/pAC compatibilized and uncompatibilized blends extruded at 197°C

Entry	Young's Modulus (GPa)	Stress at break (MPa)	Elongation at break (%)	Impact resistance (kJ/m^2)
**PLA85/pAC15**	4.3 ± 1.3	61.0 ± 2.7	2.1 ± 0.6	3.4 ± 0.5
**PLA85/pAC15_C1**	3.1 ±0.3	63.0 ± 2.6	2.6 ± 0.5	4.1 ± 0.5
**PLA85_C1**	2.6 ± 0.4	57.1 ± 1.2	2.7 ± 0.4	3.7 ± 0.5
**PLA80/pAC20**	4,4 ± 0.6	58.4 ± 2.7	1.8 ± 0.2	5.3 ± 0.4
**PLA80/pAC20_C1**	3.6 ± 0.7	57.9 ± 3.3	2.1 ± 0.5	3.8 ± 0.5
**PLA80_C1**	4.6 ± 0.3	53.2 ± 2.9	1.9 ± 0.4	2.9 ± 0.2
**PLA75/pAC25**	4.3 ± 0.1	53.3 ± 2.0	1.8 ± 0.03	3.4 ± 0.5
**PLA75/pAC25_C1**	4.1 ± 0.4	65.1 ± 4.3	2.2 ± 0.3	4.1 ± 0.5
**PLA75_C1**	3.1 ± 0.3	55.6 ± 0.8	1.8 ± 0.09	3.7 ± 0.6

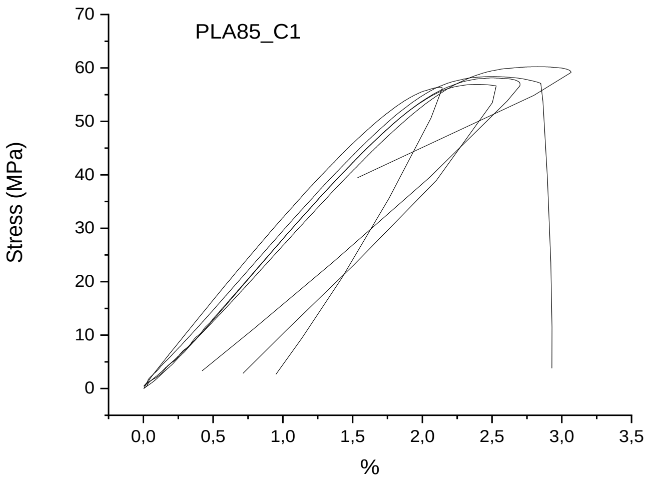

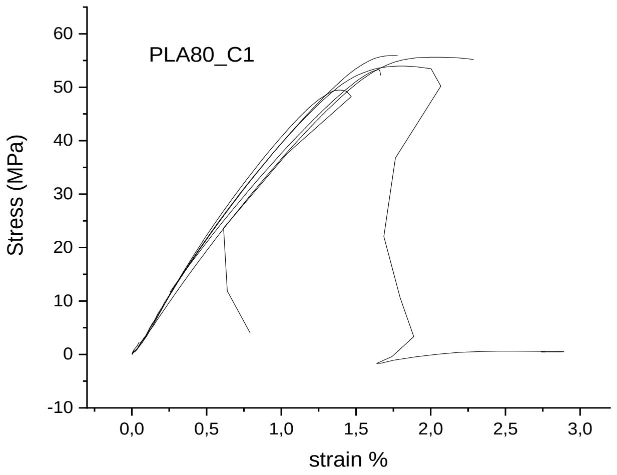

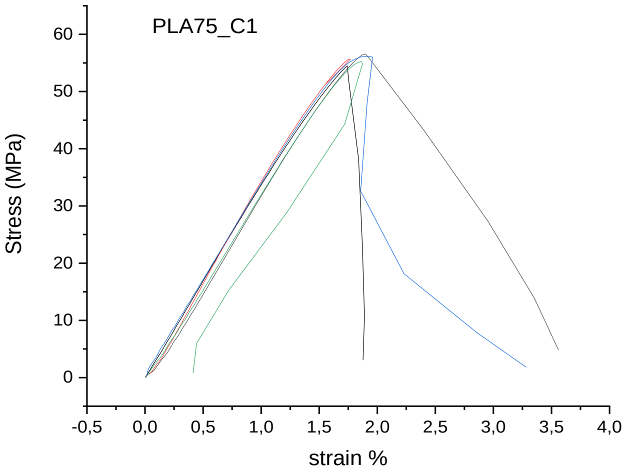

Figure 1: Overlay of 5 Stress Strain curves of PLAX_C1 blends (for calculation of Young's Modulus)

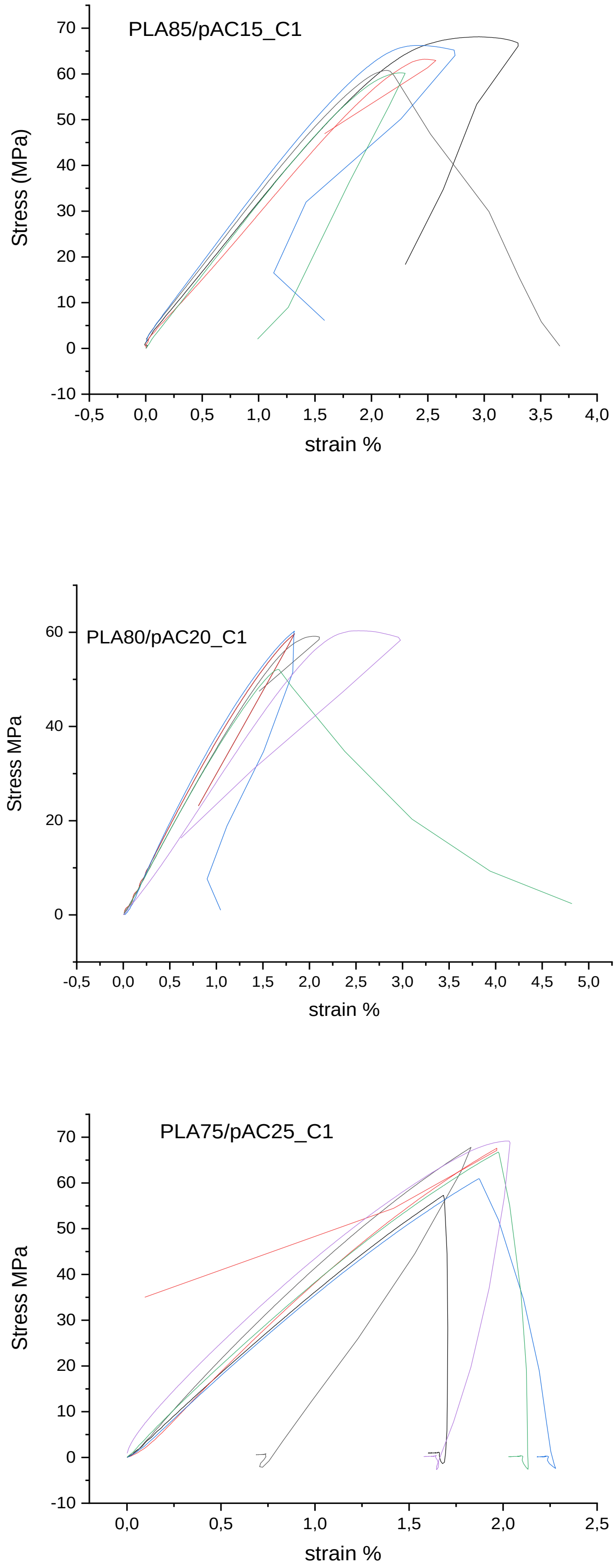

Figure 2: Superposition of 5 Stress Strain curves of PLAX_pAC(100-X)_C1 blends (for calculation of Young's Modulus)

Table 3: tensile data of each tested specimen and average values of tensile properties with standard deviation

PLA85/pAC15_Clab1 (T=197°C)			
**Specimen**	**E (GPa)**	**Elong.%**	**Stress (MPa)**
**1**	3,158734	2,260823	60,49562
**2**	2,940424	1,561389	48,23473
**3**	3,408428	3,217072	60,62666
**4**	3,935895	2,145037	60,82285
**5**	2,578572	1,762048	51,89667
**Average**	3,204411	2,189274	56,415306
**St. Dev.**	**0,509937**	**0,640353**	**5,940345191**

PLA75/pAC25 (T=230°C)			
**Specimen**	**E (GPa)**	**Elong.%**	**Stress (MPa)**
**1**	2,21	1,845	48,14
**2**	3,96	1,28	45,374
**3**	3,985	1,457	53,141
**4**	2,893	1,68	51,937
**5**	4,16	1,17	42,235
**Average**	**3,4416**	**1,4864**	**48,1654**
**St. Dev.**	**0,851164**	**0,27823066**	**4,526139779**

PLA75/pAC20_C1 (T=230°C)			
**Specimen**	**E (GPa)**	**Elong.%**	**Stress (MPa)**
**1**	3,873	1,683	57,309
**2**	4,617	1,821	67,761
**3**	3,686	1,967	67,588
**4**	3,695	1,871	60,922
**5**	4,212	1,975	66,755
**6**	4,821	2,021	69,16188
**Average**	**4,150667**	**1,889667**	**64,91614667**
**St. Dev.**	**0,483988**	**0,125128**	**4,700244926**

PLA75/pAC20_C2 (T=230°C)			
**Specimen**	**E (GPa)**	**Elong.%**	**Stress (MPa)**
**1**	2,7	2,132	61,169
**2**	5,077	1,41	54,074
**3**	4,249	1,645	54,591
**4**	4,43	1,729	58,247
**5**	6,482	1,401	56,63
**6**	3,877	1,441	49,531
**Average**	**4,469167**	**1,626333**	**55,707**
**St. Dev.**	**1,261077**	**0,282316**	**3,979751098**

PLA75/pAC25_C1 (T=197°C)			
**Specimen**	**E (GPa)**	**Elong.%**	**Stress (MPa)**
**1**	3,986000	2,6528	68,67632
**2**	4,448	2,338639	68,64668
**3**	4,294	2,109409	60,85884
**4**	4,176	1,791988	60,06512
**5**	3,360	2,161029	67,00855
**Average**	**4,0528**	**2,210771**	**65,051102**
**St. Dev.**	**0,422485**	**0,316172**	**4,25256001**

PLA80/pAC20_C1 (T=197°C)			
**Specimen**	**E (GPa)**	**Elong.%**	**Stress (MPa)**
**1**	3,904591	2,156046	59,19711
**2**	3,399405	2,061611	59,62534
**3**	3,452452	1,957505	55,34641
**4**	3,377273	2,371514	57,22602
**5**	3,750252	1,919240	52,31165
**Average**	**3,576795**	**2,08835**	**57,941306**
**St. Dev.**	**0,660705**	**0,46906**	**3,293158178**

PLA85/pAC15_C1 (T=197°C)			
**Specimen**	**E (GPa)**	**Elong.%**	**Stress (MPa)**
**1**	3,026886	3,30036	66,06518
**2**	3,15665	2,130882	60,76986
**3**	2,700989	2,57043	62,92920
**4**	3,388634	2,732482	65,20526
**5**	3,124386	2,299261	60,14073
**Average**	**3,08**	**2,61**	**63,02**
**St. Dev.**	**0,24978**	**0,45255**	**2,61773**

PLA75_C1 (T=197°C)			
**Specimen**	**E (GPa)**	**Elong.%**	**Stress (MPa)**
**1**	3,321434	1,741783	54,48643
**2**	2,778715	1,900197	56,55118
**3**	3,137050	1,762568	55,68648
**4**	3,342299	1,959666	55,99953
**5**	2,906334	1,865058	55,23692
**Average**	**3,097166**	**1,845854**	**55,592108**
**St. Dev.**	**0,249902**	**0,092253**	**0,78156897**

PLA80_C1 (T=197°C)			
**Specimen**	**E (GPa)**	**Elong.%**	**Stress (MPa)**
**1**	4,441197	1.648774;	53,26400

**2**	4,694951	1,778514	55,89730
**3**	4,058204	2,3041315	55
**4**	4,832423	14,594419	48,28285
**5**	4,736426	2,002116	53,47184
**Average**	**4,5526402**	**5,1697952**	**53,17544276**
**St.Dev**	**0,311927372**	**6,286773**	**2,942391532**

**PLA85_C1 (T=197°C)**			
**Specimen**	**E (GPa)**	**Elong.%**	**Stress (MPa)**
**1**	2,365165	2,698151	56,60642
**2**	2,430886	2,529520	56,65311
**3**	3,280148	2,142074	56,17992
**4**	2,346538	3,066647	59,15303
**5**	2,719274	2,849526	57,10575
**Average**	**2,628402**	**2,657184**	**57,139646**
**St. Dev.**	**0,393949**	**0,349274**	**1,172282529**

**PLA75/pAC25 (T=197°C)**			
**Specimen**	**E (GPa)**	**Elong.%**	**Stress (MPa)**
**1**	4,162	1,769	51,958
**2**	4,374	1,8	55,146
**3**	4,315	1,73	55,819
**4**	4,438	1,76	51,726
**5**	4,168	1,769	51,958
**Average**	**4,2914**	**1,7656**	**53,3214**
**St. Dev.**	**0,123332**	**0,025026**	**1,98935864**

**PLA80/pAC20 (T=197°C)**			
**Specimen**	**E (GPa)**	**Elong. %**	**Stress (MPa)**
**1***	3,377041	2,0103	58,71088
**2**	4,891032	1,445464	55,20692
**3**	4,542285	1,712613	59,12069
**4**	3,785144	1,832287	57,86678
**5**	4,898429	1,758027	55,28189
**6**	4,448295	2,018057	62,8307
**7***	4,567814	1,831255	60,01155
**Average**	**4,358577**	**1,801143286**	**58,43277286**
**St.Dev.**	**0,570492**	**0,19531134**	**2,678289608**

**PLA85/pAC 15 (T=197°C)**			
**Specimen**	**E (GPa)**	**Elong.%**	**Stress (MPa)**
**1**	4,330595	1,972353	64,13213
**2**	4,416047	2,99107	61,5302
**4**	3,318862	2,254742	62,64526
**6**	5,095346	1,729793	60,8335

**7**	5,28996	1,375656	56,19628
**8**	3,31852	2,270468	60,39429
**Average**	**4,28883**	**2,099013667**	**60,95527667**
**St. Dev**	**1,25842**	**0,552272676**	**2,692826117**

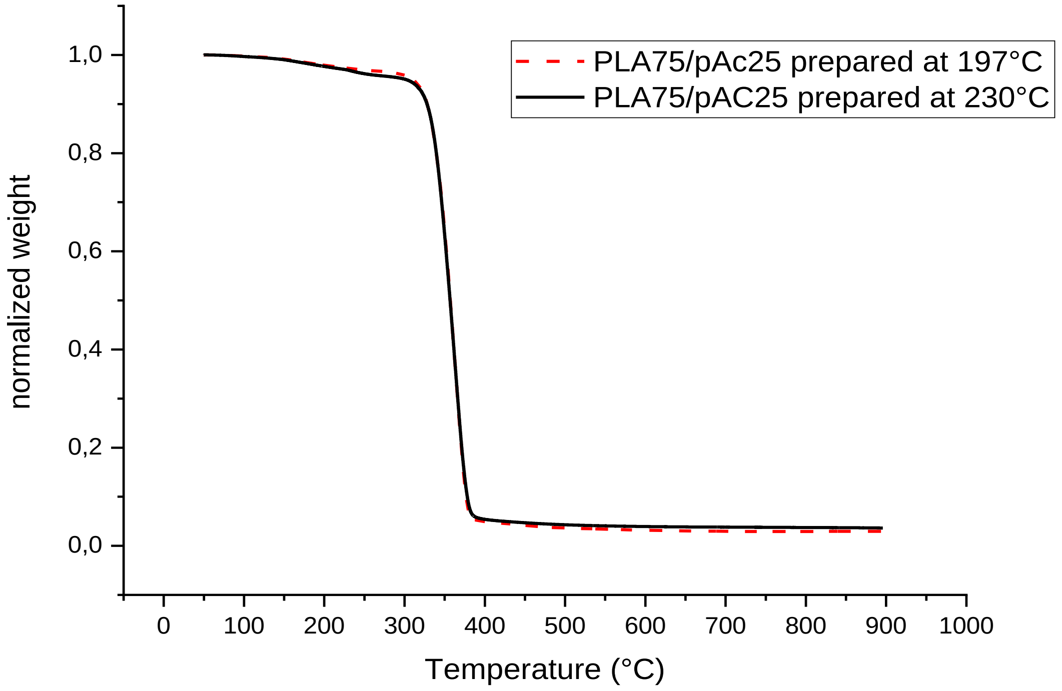

Figure 3: thermogravimetric curves obtained in Nitrogen at 10°C/min for the reference PLA75/pAC25 blend extruded at 197 and 230°C.
